# Evaluation of Phenotypic and Genotypic Characteristics of Carbapnemases-producing Enterobacteriaceae and Its Prevalence in a Referral Hospital in Tehran City

**DOI:** 10.30699/ijp.2020.111181.2188

**Published:** 2020-02-28

**Authors:** Kosar Jalalvand, Nasrin Shayanfar, Fereshteh Shahcheraghi, Elahe Amini, Mahsa Mohammadpour, Pegah Babaheidarian

**Affiliations:** 1 *Department of Pathology, Hazret-e-Rasoul Hospital, Iran University of Medical Sciences, Tehran, Iran*; 2 *Department of Bacteriology, Pasteur Institute of Iran, Tehran, Iran*; 3 *Skull Base Research Center, Hazret-e-Rasoul Hospital, The Five Senses Institute, Iran University of Medical Sciences, Tehran, Iran*; 4 *Medical student, Tehran University of Medical Sciences, Tehran, Iran*

**Keywords:** Enterobacteriacea, drug resistance, microbial, polymerase chain reaction, primer, DNA, Gene

## Abstract

**Background & Objective:**

Carbapenem-resistant Enterobacteriaceae is a growing concern worldwide including Iran. The emergence of this pathogen is worrying as carbapenem is one of the 'last-line' antibiotics for treatment of infections caused by multi drug resistant gram- negative bacteria. The main objective of this study was to determine the prevalence of carbapenem**-**resistant Enterobacteriaceae in a referral hospital in Tehran, Iran.

**Methods:**

In this study, all positive isolates of Enterobacteriaceae recorded in blood, urine, and other body fluids were studied during April 2017 to April 2018 in a referral hospital in Tehran. All cases of resistance to carbapenems were first tested by modified Hodge test. All cases with positive or negative test, after gene extraction, were examined genotypically based on the primers designed for the three Klebsiella pneumoniae carbapenemase (*KPC)*, New Delhi metallo-β-lactamase* (NDM)*, and OXA-48 genes by conventional PCR method.

**Results:**

108 isolates (13.6%) were resistant to all cephalosporins as well as to imipenem and meropenem. In a genotypic study, including 45 isolates, 13 isolates were positive for *OXA-48* gene, 11 isolates for *OXA-48* and *NDM* genes, 11 isolates for *OXA-48*, *NDM* and *KPC* genes, 4 isolates for *OXA-48* genes and *KPC*, 3 isolates for *NDM*, one isolate for *KPC*. On the other hand, two isolates were negative for all three genes examined.

**Conclusion:**

*OXA-48* gene was one of the most common genes resistant to carbapenems in Iran. According to studies, the prevalence of antibiotic resistance in Iran is rising dramatically, which reduces the choice of antibiotics to treat severe infections in the future.

## Introduction

Emergence of carbapenem-resistant *Enterob-acteriaceae *is being reported increasingly worldwide and is becoming an important issue in health care systems (1).

Nosocomial infections, a major public healthcare problem, are more prevalent in developed countries because of the related mortality and socioeconomic costs (2). *Enterobacteriaceae *is one of the major causative agents of nosocomial infections (3,4). 

Gram-negative bacteria of the Enterobacteriaceae family are important causes of urinary tract infections (UTIs), bloodstream infections, hospital- and healthcare-associated pneumonias, and various intra-abdominal infections (5).

Emerging resistance in Enterobacteriaceae is a significant problem which requires immediate attention. (5).

The carbapenem group (e.g., imipenem, meropenem) is a safe and generally effective therapeutic choice for the treatment of severe gram-negative bacterial infections when resistance to other classes of antimicrobials is present (6). Due to their broad-spectrum activity, these antibiotics are commonly used in the treatment of life-threatening infections. Excessive and inappropriate use of these drugs, has led to an increased resistance to carbapenems. This issue is one of the main causes of the expression of carbapenemase genes such as *IMP*, *VIM*, *NDM*, *OXA-48*, and *KPC* among the members of this family (7).

The production of acquired carbapenemase significantly limits the choice of antibiotic treatment infections caused by Gram-negative bacteria (8).

More seriously, Enterobacteriaceae with carba-penem-hydrolyzing-lactamases are an emerging problem (9). *OXA-48* carbapenemase (Ambler class D) was first reported in Enterobacteriaceae in Turkey in 2001 (10,11). 

Subsequently, spread has been reported not only across Turkey but also in the Middle-East, North Africa, and Europe (12). The *KPC* constitutes the most common class A carbapenemase in *Klebsiella pneumoniae* which was first reported in 1996 in the USA, though current reports reveal their presence in Europe, Asia, and South America (13).

New Delhi Metallo-beta-lactamase was first reported in K. pneumonia and *Escherichia coli* isolated from a 59-year-old Swedish patient from India who was previously admitted to a hospital in New Delhi (14). The main objective of this study is to show the prevalence of carbapenem-resistant Enterobacteriaceae and to investigate the presence of carbapenemase genes in Enterobacteriaceae clinical isolates resistant to carbapenem antibiotics from hospitalized patients in a university referral hospital in Tehran, Iran.

## Materials and Methods

**Clinical Isolates**

A total of 800 Enterobacteriaceae isolates were collected from various clinical specimens including urine (n=473), tracheal aspirate (n=19), broncho alveolar-lavage (BAL) fluid (n=11), wound (n=44), cerebrospinal fluid (n=2), sputum (n=78), catheter (n=9), other sterile body fluid (n=99), and blood ( n=63) between April 2017 and April 2018 from hospitalized patients at Rasoul Akram hospital, one of the referral university hospitals of Tehran, Iran.

**Bacterial Isolation and Identification**

All samples were routinely cultured on MacConkey and blood agar plates. Blood samples were cultured in Blood culture bottles. The isolates were identified at the species level using standard biochemical tests and microbiological methods such as colony types, motility, carbohydrate fermentation of glucose, lactose and sucrose, Triple Sugar Iron (TSI), Simmons citrate, SIM (Sulfide, Indole, Motility), Methyl Red, and urease. These are common biochemical tests used for identifying different species of Enterobacteriaceae (15).

**Antibiotic Susceptibility Test**

The tested antibiotics were amikacin and gentamicin (aminoglycosides); ampicillin-sulbactam, and piperacillin-tazobactam (β-lactams); cephazolin, cefepime, cefotaxime, ceftazidime, (cephalosporins); ciprofloxacin (quinolone); Sulfamethoxazole / Trimethoprim; colistin (polymyxin), imipenem and meropenem (carbapenems) and Nitrofurantoin (in urinary infection) (MAST company, Germany). They were investigated using the disc-diffusion method as described in Clinical and Laboratory Standards Institute (CLSI) guidelines (16). We used *E. coli* ATCC 25922 as a carbapenem-susceptible strain, and *K. pneumoniae* AO 8053 as a carbapenem resistant for quality control. MIC to meropenem was determined by E-test strips (Liofilchem company, Italy), according to the manufacturer’s instructions and CLSI 2015 guidelines. The isolates with MIC values ≥4 μg/mL ;2 μg/ mL and ≤1 μg /mL for meropenem were considered as resistant, intermediate, and susceptible, respectively (16).

Phenotypic determination of carbapenemases was performed using the modified Hodge test (MHT). The MHT was carried out according to CLSI guidelines using a 10μg disc of ertapenem on Muller–Hinton agar plates. Following overnight incubation, the presence of a “cloverleaf shaped” inhibition zone was interpreted as a positive result. *E. coli* ATCC 25922 was used as the carbapenem susceptible strain (17).

**Molecular Detection of Genes Encoding Carbapenemases**

Genomic DNA isolates was obtained from Entrobacteriaceae by boiling two or three colonies of each isolate in 500 mL of distilled water for 10 min and centrifugation at 10,000 rpm for 10 min. The supernatant was used as a template for polymerase chain reaction (PCR) assay. PCR assays were carried out for detection of *KPC*, *NDM*, and *OXA-48* genes using a set of specific primers as previously described (Table 1) (2,18–21). Standard strains for *blaOXA-48*, *blaNDM*, and *blaKPC* genes were provided by the Pasteur Institute of Iran. The study was approved by the ethics committee of the Iran University of Medical Sciences with the (code: IR.IUMS.FMD.REC.1396.9411100003 ). 

**Table 1:** The sequencing primers used in this study

Gene	3ˊ–5ˊ Primer	Product size (bp)	Annealing Temperature (C)	References
***KPC-Fm***	CGTCTAGTTCTGCTGTCTTG	798	60	(2)
***KPC-Rm***	CTTGTCATCCTTGTTAGGCG		60	(2)
***NDM-F***	GGTTTGGCGATCTGGTTTTC	621	55	(2)
***NDM-R***	CGGAATGGCTCATCACGATC		55	(2)
***OXA-48-F***	GCGTGGTTAAGGATGAACAC	438	55	(2)
***OXA-48-R***	CATCAAGTTCAACCCAACCG		55	(2)

**Statistical **A**nalysis**

Data was presented as mean and standard deviation for quantitative variables, and percentage for qualitative variables. The correlation between the variables was assessed by Pearson correlation test**. **Mann-Whitney U test or t-test was applied to compare quantitative variables. SPSS 22 (IBM Corp., Armonk, NY, USA) was used to analyze the statistical variables. P-values less than 0.05 were set as statistically significant level

## Results

***Bacterial Isolates***

Here, 108 (13.6%) of 800 Enterobacteriaceae isolates (481 *E. coli*, 291 *K. pneumoniae*, 8 Enterobacter and 20 other Enterobacteriaceae( collected were carbapenem-resistant Enterobacteriaceae (CRE) consisting of *K. pneumoniae* (n=102), *E. coli* (n=4), and Enterobacter (n=2). Carbapenem-resistant was 35% in *K. pneumoniae*, 25% in Enterobacter and less than 1% in *E. coli* isolates.

A total of 72 isolates (66.66%) were isolated from ICU wards, while the remaining isolates were recovered from other wards. The isolates were predominantly collected from sputum (29/108; 27%), urine (24/96; 25%), wound secretions (10/108; 9.5%), %), broncho alveolar lavage (BAL) (9/108; 8.5%), catheter (3/108; 3%), tracheal secretions (2/108; 1.85%), CSF (1/108; 1%), and other sites of isolation (18/108; 16%). Most carbapenem-resistant *K. pneumoniae* isolates were obtained from respiratory specimens including sputum, tracheal secretions, and BAL (39/102; 38%) and urine specimens (22/102; 21.5%). 

Both carbapenem-resistant Enterobacters were isolated from blood cultures, separated from the same ICU ward. Carbapenem-resistant *E. coli* were isolated from urine (2/4;50%), sputum (1/4; 25%) and wound secretion (1/4; 25%) specimen. Interestingly, three of the four isolates were found in the same ICU ward.

 The age of patients ranged from 16 years to 91 years (mean = 58.53 years). Male-to-female ratio was 1.66 and 59% of patients were over 60 years old. The isolates from intensive care units covered 67% of carbapenem-resistant Enterobacteriaceae, which included 100% of *E. coli* and Enterobacter but 65% of *K. pneumoniae*.

**Antimicrobial Susceptibility Testing**

Overall, all isolates were resistant to at least three classes of antibiotics and they were considered as MDR. The rates of resistance to imipenem and meropenem among isolates were 90% (97/108) and 96.4% (104/108), respectively. The percentages of resistance to other antimicrobial agents were as follows: colistin 5.5% (6/108); trimethoprim /sulfamethoxazole 80% (86/108); amikacin 89% (96/108); and nitrofurantoin 71.5% (17/24). All isolates demonstrated resistance to ceftazidime, cefepime, cefazolin, cefotaxime, ciprofloxacin, ampisulbactam-piperacillin.

Of the 45 carbapenem resistant *entrobacteriaceae* isolates, all were high-level meropenem-resistant (MIC ≥ 4), except for 4 isolates. The lowest and highest level of meropenem MIC for carbapenem-resistant isolates were estimated 0.16 mg/L and >32 mg/L, respectively. 

The results of MHT revealed that 33 of 45 carbapenem-resistant isolates were carbapenemase producing ones.

**Detection of Carbapenemases**

**Fig.1:**
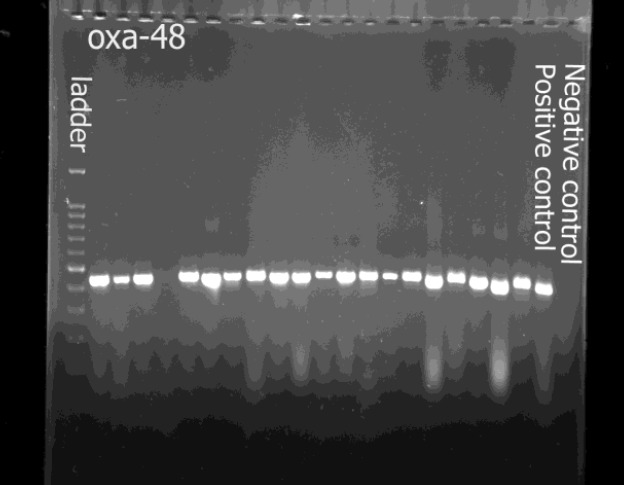
PCR product of blaOXA-48 gene

Forty-five isolates were included in the molecular study as CRE. Overal, of these 45 CREs, 13 (28.88%) harbored only the *OXA-48* gene; 3 (6.66%) carried only the *NDM* gene, and 1 (2.22%) carried only the *KPC* gene. Further, 11 (24.44%) of these isolates co-produced the *NDM* and *OXA-48* genes; 4 (8.8%) co-produced the *OXA-48* and *KPC*; and 11 (24.44%) co-produced the *NDM*, *OXA-48*, and *KPC* genes. Two isolates were negative for all 3 genes. There was no statistical association between the source of infections and gene expression (*P*=0.32). The results of tests performed in Carbapenem producing Entero-bacteriaceae are summarized in Table 2. Figure 1 shows a PCR product of *blaOXA-48* gene. The first column represents the ladder and the last two columns represent positive and negative controls.

**Table 2:** Characteristics of carbapenem producing Enterobacteriaceae

Isolates	Age/Gender	MIC	MHT	Genes	Ward	Specimen	Outcome
**1(** ***K. pneumonia*** **)**	27/Female	>32	positive	OXA48+NDM+KPC	SICU	Tracheal	Survived
**2(** ***K. pneumonia*** **)**	67/Female	>32	positive	OXA48+NDM1+KPC	ICU	tracheal	Died
**3(** ***K. pneumonia*** **)**	54/Female	>32	positive	OXA48+NDM+KPC	Post CCU	pancreas	Survived
**4(** ***K. pneumonia*** **)**	56/Male	>32	positive	OXA48+KPC	SICU	Sputum	Survived
**5(** ***K. pneumonia*** **)**	68/Male	>32	positive	OXA48+NDM+KPC	MICU	Sputum	Died
**6(** ***K. pneumonia*** **)**	47/Male	>32	positive	OXA48+NDM+KPC	Neuro ICU	Unknown	Died
**7(** ***K. pneumonia*** **)**	62/Femal	>32	positive	OXA48+NDM+KPC	SICU	Blood	Died
**8(** ***K. pneumonia*** **)**	21/Femal	>32	positive	NDM1	Gynecology	Urine	Survived
**9(** ***K. pneumonia*** **)**	78//Femal	>32	positive	OXA48+NDM	MICU	Sputum	Died
**10(** ***K. pneumonia*** **)**	80/Femal	>32	positive	OXA48+NDM	MICU	Sputum	Died
**11(** ***K. pneumonia*** **)**	65/Femal	>32	positive	OXA48+NDM+KPC	MICU	Urine	Survived
**12(** ***K. pneumonia*** **)**	23/Male	12	Negative	OXA48	MICU	Sputum	Died
**13(** ***K. pneumonia*** **)**	62/Male	>32	Negative	Non	MICU	Sputum	Died
**14(** ***K. pneumonia*** **)**	35/Male	>32	positive	OXA48+NDM+KPC	ICU	CVP	Survived
**15(** ***K. pneumonia*** **)**	72/Male	>32	positive	OXA48+NDM+KPC	MICU	Sputum	Died
**16(** ***K. pneumonia*** **)**	56/Male	>32	positive	OXA48+NDM	SICU	Wound secretion	Died
**17(** ***K. pneumonia*** **)**	55/Male	>32	Negative	Non	MICU	Sputum	Died
**18(** ***K. pneumonia*** **)**	64/Male	>32	positive	OXA48+NDM	MICU	Sputum	Survived
**19(Entrobacter)**	73/Male	>32	positive	OXA48+NDM	ICU	Blood	Died
**20(** ***K. pneumonia*** **)**	62/Male	>32	positive	OXA48+NDM+KPC	MICU	Urine	Died
**21(** ***K. pneumonia*** **)**	50/Male	0.16	positive	OXA48	SICU	Wound secretion	Survived
**22(Entrobacter)**	71/Female	>32	positive	OXA48+NDM	ICU	Blood	Died
**23(** ***K. pneumonia*** **)**	60/Male	>32	positive	OXA48+NDM+KPC	MICU	Blood	Died
**24(** ***K. pneumonia*** **)**	73/Male	>32	Negative	OXA48	ICU	Sputum	Died
**25(** ***K. pneumonia*** **)**	70/Female	>32	Negative	OXA48	ICU	Sputum	Died
**26(** ***K. pneumonia*** **)**	84/Female	>32	positive	OXA48	Nephrology	Urine	Survived
**27(** ***K. pneumonia*** **)**	46/Male	0.16	positive	OXA48	Infectious	Catheter	Survived
**28(** ***K. pneumonia*** **)**	56/Male	0.16	positive	OXA48	SICU	Wound secretion	Died
**29(** ***K. pneumonia*** **)**	71/Female	6	Negative	OXA48	MICU	Pleura Fluid	Died
**30(** ***K. pneumonia*** **)**	75/Female		positive	KPC	SICU	Ascites fluid	Died
**31(** ***E. coli*** **)**	75/Male	6	Negative	OXA48	Neuro ICU	Urine	Died
**32(** ***E. coli*** **)**	29/Male	4	positive	OXA48+KPC	Neuro ICU	Urine	Survived
**33(** ***K. pneumonia*** **)**	74/Female	4	positive	OXA48+KPC	SICU	Urine	Died
**34(** ***K. pneumonia*** **)**	36/Female	3	Negative	OXA48	Medical	Sputum	Survived
**35(** ***K. pneumonia*** **)**	37/Male	6	Negative	OXA48	ICU	Unknown	Died
**36(** ***K. pneumonia*** **)**	67/Female	4	positive	OXA48+KPC	Surgery	Wound secretion	Survived
**37(** ***K. pneumonia*** **)**	50/Male	4	Negative	OXA48	Infectious	Wound secretion	Survived
**38(** ***K. pneumonia*** **)**	40/Female	32	Negative	NDM	Neuro ICU	Sputum	Died
**39(** ***K. pneumonia*** **)**	38/Male	32	Negative	NDM	MICU	Urine	Died
**40(** ***K. pneumonia*** **)**	77/Male	6	positive	OXA48+NDM	ICU	Sputum	Died
**41(** ***K. pneumonia*** **)**	84/Female	>32	positive	OXA48+NDM	Nephrology	Urine	Died
**42(** ***K. pneumonia*** **)**	89/Male	>32	positive	OXA48+NDM	Medical	Urine	Survived
**43(** ***K. pneumonia*** **)**	61/Male	>32	positive	OXA48+NDM	Medical	Urine	Survived
**44(** ***K. pneumonia*** **)**	42/Male	>32	positive	OXA48+NDM	Neuro ICU	Sputum	Died
**45(** ***K. pneumonia*** **)**	48/Male	>32	positive	OXA48	SICU	Unknown	Survived

## Discussion

Antibiotic resistance, especially simultaneous resistance to multiple classes of antibiotics (multiresistance), is an increasing global problem. Gram-negative bacteria, in particular the Enterobacteriaceae, are adapted to exchanging genetic information and antibiotic resistance. In these organisms, this is often due to the acquisition of genes from a shared pool (22). Resistance to carbapenems amongst Enterobacteriaceae is usually mediated by transferable beta-lactamase enzymes. Due to frequent occurrence of other resistance genes on the same mobile genetic elements, strains producing carbapenemases are normally extensively drug resistant (23).

 The emergence of carbapenemases in Enterobacteriaceae is of great concern as these provide a far more efficient and stable mechanism of resistance to carbapenems than combinations of an ESBL and impermeability. Bacteria carrying these resistance determinants are often resistant to other treatment options, due to the frequent co-acquisition of non-beta-lactam resistance genes located on the same mobile genetic elements. Further, acquired carbapenemases have the potential for sepsis (23,24). Although the presence of CRE have repeatedly been documented, larger scale, systematic studies with molecular investigations are still few in Iran (25). The prevalence of CRE in 2017-2018 collected at Rasoul Akram hospital was 13.6% in general. Specifically, the prevalence of carbapenems resistance in *K. pneumoniae* was 35%; in Enterobacter 25%, and in* E. coli* less than 1%. On the other hand, in Solgi *et al.*’s study in 2015-2016 done in Isfahan, prevalence of CPKP was 41.7% (20). In this study, two Enterobacter isolates were obtained from the same ICU ward and from blood culture of patients at the same time. Also their gene presence was identical. 3 out of 4 carbapenem-resistant *E. coli* cases were obtained from the same ICU ward. They were all isolated from urine samples. Their gene presence was also identical. All instances of carbapenem resistance Entrobacteriaceae in the MICU ward were *K. pneumoniae*, and their gene presence was also similar except for one. This demonstrates the importance of infection control in hospital wards.

 The highest number of clinical isolates was collected from the respiratory tract and in the MICU. The highest antibiotic resistance was observed for cephalosporins and carbapenems while the least resistance to colistin and trimethoprim/ sulfamethoxazole. In the present study, the rate of antibiotic resistance with some variation which may arise from the source of infections and geographical distribution was comparable with the previous reported studies in Iran (18,20,26,27). The administration of multiple antibiotics has been reported as a risk factor for carbapenem resistance acquisition (28). Findings by Patel *et al.* showed that carbapenem resistance is not attributed only to previous exposure to carbapenem but also exposure to other antibiotics (29). In our study, the ratio of male to female was 1.66, while in Kuwait, Arabian Peninsula, Malaysian studies, the male to female ratios were 0.9, 1.8, and 1.2, respectively. (30–32). In our study, 52.77% of the resistant group died, which was significantly (*P*=0.043) in contrast to the carbapenem-sensitive group, where only 12.87% died. In the Kuwait study, 71.42% of the patients survived while 28.57% died (30). Dautzenberg and co-workers have reported that patients colonized with carbapenem resistant Enterobacteriaceae have higher mortality rates as compared to non-colonized patients due to long hospitalization (33). These results are consistent with the results of our study. Risk factors for infection with carbapenemase- producing Enterobacteriaceae (CPE) include hospitalization (particularly in intensive care units), mechanical ventilation, indwelling catheters, comorbidities, transplantation, exposure to broad-spectrum antibiotics and previous colonization with these strains (23). This may be the cause of higher mortality in this group and requires further investigation. Considering the frequency of isolates resistant to carbapenem, according to the hospitalization wards, in our study, 67% of isolates were isolated from intensive care units. Similarly, in Solgi's study in Isfahan, 77% of the cases were isolated from intensive care units (20). However, in the Malaysian study, the highest percentages were observed in the surgical ward (25%) and internal ward (25%), some of whom were also admitted to the intensive care unit (32).

Our data regarding strains collected in one of referral hospital in Tehran is in accordance with previous reports that the most common mechanism of carbapenem resistance in CRE isolates of Tehran are the production of *OXA-48*-like and *NDM* carbapenemases (20).

This study found *CPE* with *OXA-48*, *NDM* and *KPC* genes together or alone, in a hospital in Tehran. The co-production of *OXA-48*- and *NDM*; *OXA-48* and *KPC* with *NDM* -producing *K. pneumoniae* has been reported in some Asian and European countries (30–32,34–36). Beyond the dominance of *OXA-48*-like and *NDM* producing strains, it was also notable that almost half of the collection co-produced these two enzymes. Although, dual carbapenemase producing *K. pneumoniae *were encountered in Iran earlier (20). Their occurrence with such frequency has not been noted yet. To the best of our knowledge, this report presented the first identification of *OXA-48* and *KPC* with *NDM* -producing *K. pneumoniae* in Iran.

Genes of the *OXA-48* type and related OXA enzymes have been found to be widely prevalent in North Africa, the Middle East, and the Indian subcontinent (12). And, more importantly, large numbers of outbreaks have occurred in regions such as Europe and Australia, where CRE is not endemic, as a result of international transfer of patients (12,37). The first report of *OXA-48* gene in Iran was by Azimi *et al.* in Tehran 2014, in 27 carbapenem-resistant *K. pneumoniae* isolates recovered from burn patients (1). It was subsequently re-identified in Iran (20,27). The widespread prevalence of *OXA-48*-positive carbapenem resistant *K. pneumoniae* strains has been reported in several Asian and European countries including Turkey, Saudi Arabia, Taiwan, China, Russia and France which has become an expanding problem (11,32,38–42).

A majority of *NDM* cases reported worldwide were related to travel or hospitalization in the Indian subcontinent such as India and Pakistan (9). However, in our study, the NDM positive patient had no record of prior travel outside Iran. The presence of *blaNDM* without any association with international travel has also been reported by Rimrang and coworkers (43) which indicated that the *NDM* gene was acquired locally. The first report on the detection of NDM in Iran was by Shahcheraghi *et al.* in 2012 during a study on *Enterobacteriaceae *family isolates collected from five hospitals in Tehran (18). NDM was also reported by Fazeli and Solgi *et al.* (44,45). KPC is endemic in northeastern regions in the USA, Greece and Israel but cross regional spreading into United Kingdom, Brazil, Sweden, India and China had been reported recently (46). The first report of *KPC* in Iran was made by Nobari and his colleagues in 2012 (21). This gene is often detected on mobile genetic elements such as plasmids and transposons which facilitates its rapid dissemination worldwide (47).

Regarding the prevalence of carbapenemase resistant genes in our study, the most frequent genes were *OXA-48* in 13 cases (28.88%), co-produced *OXA-48* and *NDM* in 11 cases (24.44%), simultaneous expression of co-produced *NDM*, *OXA-48*, and *KPC* In 11 cases (24.44%), co-produced *OXA-48* and *KPC* in 4 cases (8.88%), *NDM* in 3 cases (6.66%), and *KPC* in one case (2.2%), revealing that the highest frequency belonged to *OXA-48*. *OXA-48* (58.3%) and the co-produced *OXA-48* and *NDM* in 35.4% of cases have been reported in Solgi's study in Isfahan. However, no case of *KPC* was reported (20). Only clinical isolates were included in our study whereas the study By Solgi *et al.* was focused on rectal swabs (48). *OXA-48* expression in 37.5% of cases and co-produced *OXA-48* and *KPC* were also found in 37.5% of cases, with one case harboring *KPC* gene in the Malaysian study (32).

However, in the Arabian Peninsula, the *NDM* gene was found in 46.5% of cases and *OXA-48* in 32.5% of cases while their co-produced version was reported in 3.5% of cases (31). In the Kuwait study, *NDM* gene was reported in 57.1% of isolated and the co-produced version of the three genes and that of *NDM* and *OXA-48* was reported in 33.33% and 9.52% of cases, respectively (30). Based on Solgi's study, they found that intestinal carriage rates of *NDM* and *OXA-48*- producing Enterobacteriaceae are high, suggesting that *NDM* and *OXA-48* have become endemic (48). Additionally, recent observations in neighboring countries, such as Kuwait (49) and Lebanon (50), indicates the emergence of this resistance mechanism in the Middle East. We could not find any of the targeted genes in the 2 carbapenem-resistant isolates, so their resistance to carbapenems may be due to other mechanisms including production of extended-spectrum beta-lactamases (ESBLs), AmpC betalactamases, decreased permeability of outer membrane or efflux pumps activity, or probably the presence of other genes that were not studied in this research (51) .Concurrent presence of carbapenemic genes in bacteria causes many concerns for physicians in the hospital as they cause hydrolysis of all β-lactam antibiotics as an important treatment option. Furthermore, the transfer of these genes among carriers and patients admitted in hospital increases the prevalence of the disease, which is difficult to control (52,53).

This study is limited by its small sample size as it only includes the carbapenem resistant Entrobacteriacae which were isolated during a 12 months period. Thus, the findings cannot be generalized to a broader population based on this study alone as it may include potential biases. A further limitation of our study is the high number of unknown or missing values in the analysis of potential risk factors. This reflects the difficulty to obtain information such as, previous hospitalization and travel history since microbiological laboratories often have no direct access to these data.

The present study suggested that the isolates of carbapenemase-producing Enterobacteriaceae have a high prevalence in patients admitted to our hospital. The presence of these genes upon transference of one strain to another strain, rapid detection of carbapenemase-producing isolates in clinical specimens, as well as rapid and accurate screening of patient carriers at the first stage of admission to hospitals to prevent dissemination of these strains are very important at the both hospital and community level. Undoubtedly, the emergence and release of these genes in the future will reduce the choice of antibiotics suitable for the treatment of severe infections. Ultimately, this will seriously threat the human health and healthcare system of the country. Therefore, it is imperative that a continuous monitoring system and strong programs to control the hospital infection should be used to prevent further dissemination in health centers and in the community. Otherwise, one of the biggest problems we face in the future is the loss of an important antibiotic treatment line, carbapenem, an increase in mortality rates, and an increase in the cost of treatment.
